# Epidemiological investigation on diseases of *Larimichthys crocea* in Ningbo culture area

**DOI:** 10.3389/fcimb.2024.1420995

**Published:** 2024-06-19

**Authors:** Shengwei Xu, Mingfeng Ge, Juan Feng, Xinxian Wei, Honglian Tan, Zheng Liang, Guixiang Tong

**Affiliations:** ^1^ China (Guangxi)-ASEAN Key Laboratory of Comprehensive Exploitation and Utilization of Aquatic Germplasm Resources, Ministry of Agriculture and Rural Affairs, Nanning, China; ^2^ Key Laboratory of Aquaculture Genetic and Breeding and Healthy Aquaculture of Guangxi, Guangxi Academy of Fishery Sciences, Nanning, China; ^3^ Key Laboratory of South China Sea Fishery Resources Exploitation & Utilization, Ministry of Agriculture and Rural Affairs, Guangdong, China; ^4^ Aquatic Disease Control Department, Ningbo Ocean and Fisheries Research Institute, Ningbo, Zhejiang, China

**Keywords:** *Larimichthys crocea*, disease, co-infection, epidemiology, pathogen

## Abstract

**Introduction:**

Due to the high-density farming of *Larimichthys crocea* over the years, diseases caused by pathogens such as bacteria, viruses, and parasites frequently occur in Ningbo, posing a huge threat and challenge to the sustainable and healthy development of the *L. crocea’s* bay farming industry. In order to understand the diseases occurrence in *L. crocea* farming in Ningbo area, an epidemiological investigation of *L. crocea* diseases was carried out through regular sampling in 2023.

**Methods:**

From April to October 2023, routine sampling of *L. crocea* was conducted monthly in various farming areas in Ningbo. Each time, live or dying *L. crocea* with obvious clinical symptoms were sampled, with a total number of 55 *L. crocea* collected. The samples were preserved in ice bags and transported to the laboratory for pathogen detection(including bacterial isolation and identification,virus identification, and parasites detection).

**Results:**

A total of fifty-five fish dying *L. crocea* with obvious clinical symptoms were collected in this study, of which 78.18% (43/55) were detected with symptoms caused by pathogenic infection, while 21.82% (12/55) did not have identified pathogens, which were presumed to be breeding abrasions, nutritional metabolic disorders, unconventional pathogens infection or other reasons. A total of twenty-five pathogenic bacteria strains were isolated, which mainly were Pseudomonas plecoglossicida and *Vibrio harveyi*, accounting for 52% (13/25) and 32% (8/25) of the pathogenic bacteria strains, respectively. Among them, both *V. harveyi* and *Streptococcus*. iniae co-infected one fish. Additionally, three other bacterial strains including *Nocardia seriolae*, *Staphylococcus Saprophyticus*, and *Photobacterium damselae* subsp.damselae were isolated. Microscopic examination mainly observed two parasites, *Cryptocaryon irritans* and *Neobenedenia girellae*. In virus detection, the red sea bream iridovirus (RSIV) was mainly detected in *L. crocea*. Statistical analysis showed that among the fish with detected pathogens, 55.81% (24/43) had bacterial infections, 37.21% (16/43) had parasitic infections, and 37.21% (16/43) had RSIV infections. Among them, five fish had mixed infections of bacteria and parasites, three had mixed infections of bacteria and viruses, three had mixed infections of parasites and viruses, and one *L. crocea* had mixed infections of viruses, bacteria, and parasites.

**Discussion:**

These findings indicate that these three major types of diseases are very common in the *L. crocea* farming area in Ningbo, implying the complexity of mixed infections of multiple diseases.

## 1 Introduction

*Larimichthys crocea*, also known as the large yellow croaker, belonged to the Perciformes, Sciaenidae, and *Larimichthys*. *L. crocea* is one of the China-specific marine economic fishes with its delicious meat, rich nutrition, and high protein (Shi et al., 2023).China Fishery Statistical Yearbook shows that the aquaculture production of *L. crocea* reached 257,700 tons in 2022, making it the highest yield of marine aquaculture fish in China (Fisheries and Fisheries Administration of the Ministry of Agriculture and Rural Affairs et al., 2023).

Currently, the main cultivation models for *L. crocea* in China include bay cage farming, deep-sea (farming vessels), enclosure (block net) farming, and so on. The extensive development of deep-sea and enclosure farming models has played a positive role in the *L. crocea* industry, yet now these two farming models are still in the initial stage. Bay cage farming is still the main farming model, accounting for over 95% of the total production of *L. crocea*. Ningbo is one of the main farming areas for *L. crocea* in China. In 2023, the farming area of *L. crocea* in Ningbo reached over 200,000 m^2^, with an annual output of 4000 tons and a value of over 200 million yuan, forming a complete farming industry chain including breeding, seedling breeding, aquaculture production, and feed processing, with the main farming model of the bay farming. Due to the high-density farming over the years, the open water system has been severely damaged in Ningbo. Diseases caused by pathogens such as bacteria, viruses, and parasites frequently occur, posing a huge threat and challenge to the sustainable and healthy development of the *L. crocea*’s bay farming industry (Zhao et al., 2016; Zhou et al., 2023).

This study conducted an epidemiological investigation on the main diseases of *L. crocea* in the bay farming area of Ningbo, attempting to reveal the harm, prevalence characteristics, as well as disease patterns of related diseases in this area to *L. crocea*, providing basic information for formulating effective prevention and control measures in this breeding areas.

## 2 Materials and methods

### 2.1 Samples collection

From April to October 2023, routine sampling of *L. crocea* was conducted monthly in various farming areas in Ningbo (including Xiangshanxihu Port, Sanmen Bay, Fenghuashiyan Port, Ninghaibaishishan). Each time, live or dying *L. crocea* with obvious clinical symptoms were sampled, with a total number of 55 *L. crocea* collected. The samples were preserved in ice bags and transported to the laboratory for pathogen detection.

### 2.2 Bacterial isolation and identification

Under sterile conditions, tissues such as liver, spleen, and kidney of *L. crocea* were streaked onto the Brain Heart Infusion Broth (BHI) solid medium and cultured at 28°C for 24–48 hours (*Nocardia seriolae* needs to be cultured for 5–7 days). Pick single colonies and purified them, and then bacterial DNA was extracted. PCR amplification detection was carried out using 16S universal primers (the upstream primer sequence: 5′-AGAGTTTGATCMTGGCTCAG-3′; the downstream primer sequence: 5′-GGTTACCTTGTTACGACTT-3′). The PCR reaction system included 12.5μL Taq master Mix, 8.5μL ddH_2_O, 1μL upstream primer,1μL downstream primer, and 2μL DNA template. The PCR reaction conditions were: 94°C for 2 minutes; 94°C for 30 seconds, 60°C for 30 seconds, 72°C for 30 seconds, 35 cycles; and 72°C for 2 minutes. The amplified products were detected using 1.5% agarose gel electrophoresis and sent to Hangzhou Youkang Biotechnology Co., LTD for sequencing. Sequences were blasted with the NCBI database for bacterial identification.

### 2.3 Virus identification

Diseased *L. crocea* were examined for three types of viruses, specifically including the infectious spleen and kidney necrosis virus (ISKNV), red sea bream iridovirus (RSIV), and nervous necrosis virus (NNV). ISKNV detection was conducted according to the standard file of the People’s Republic of China Aquaculture Industry Standards SC/T 7216–2011. Specifically, the spleen and kidney tissues of *L. crocea* were collected, and the DNA extraction was performed using a genomic DNA extraction kit. Target sequence primers (**Table 1**) was used to PCR amplification, and agarose gel electrophoresis was used to verify the above-amplified products.

**Table 1:** Virus detection primer information.

No.	Primer name	Primer sequence (5’-3’)	Target sequence size	Virus
**1**	ISKNV -F	5’-CGTGAGACCGTGCGTAGT-3’	562bp	ISKNV
	ISKNV -R	5’-AGGGTGACGGTCGATATG-3’		
**2**	RSIV-F	5’-CTCAAACACTCTGGCTCATC-3’	570bp	RSIV
	RSIV-R	5’-GCACCAACACATCTCCTATC-3’		
**3**	NNV-F	5’-CGTGTCAGTCATGTGTCGCT-3’	421–430bp	NNV
	NNV-R	5’-CGAGTCAACACGGGTGAAGA-3’		

Refer to the People’s Republic of China National Standard GB/T36191–2018, RSIV was detected by collecting the tissues of the spleen, kidney, heart, intestine, and gill. After DNA extraction, PCR detection was performed with the following primer in **Table 1**. According to the standard file of the People’s Republic of China Aquaculture Industry Standards SC/T 7216–2012, the identification of NNV were executed by extracting the RNAs of brain tissues of *L. crocea*. After cDNA was obtained by reverse transcription, PCR amplification were performed with the corresponding primes (**Table 1**) and PCR products were determined by running agarose gel electrophoresis.

### 2.4 Parasites detection

Diseased *L. crocea* may be the result of a parasite infection, parasite detection is extremely necessary for taking effective measures to prevent the infection spread. The gill filaments, lesion ulceration sites, body attachments, or mucus of the sick *L. crocea* were sampled and then placed on slides with drops of sterile saline on them. After dispersing tissues with tweezers, coverslips were placed on top of tissues and slightly pressed flat, and then observed under a microscope.

## 3 Results

### 3.1 Pathogens detection statistics

**Figure 1:**
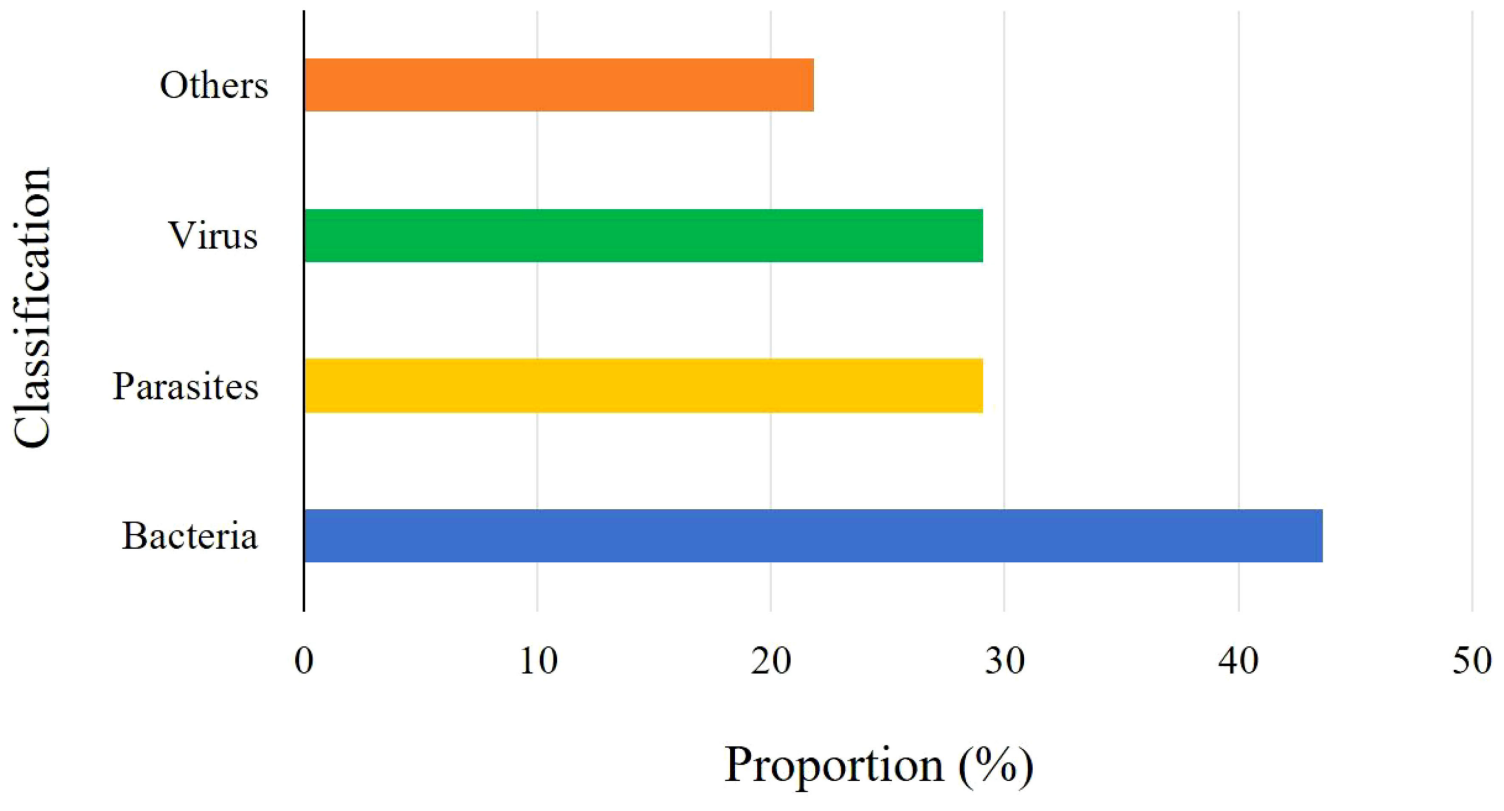
Proportion of various pathogens in *L. crocea*.

**Figure 2:**
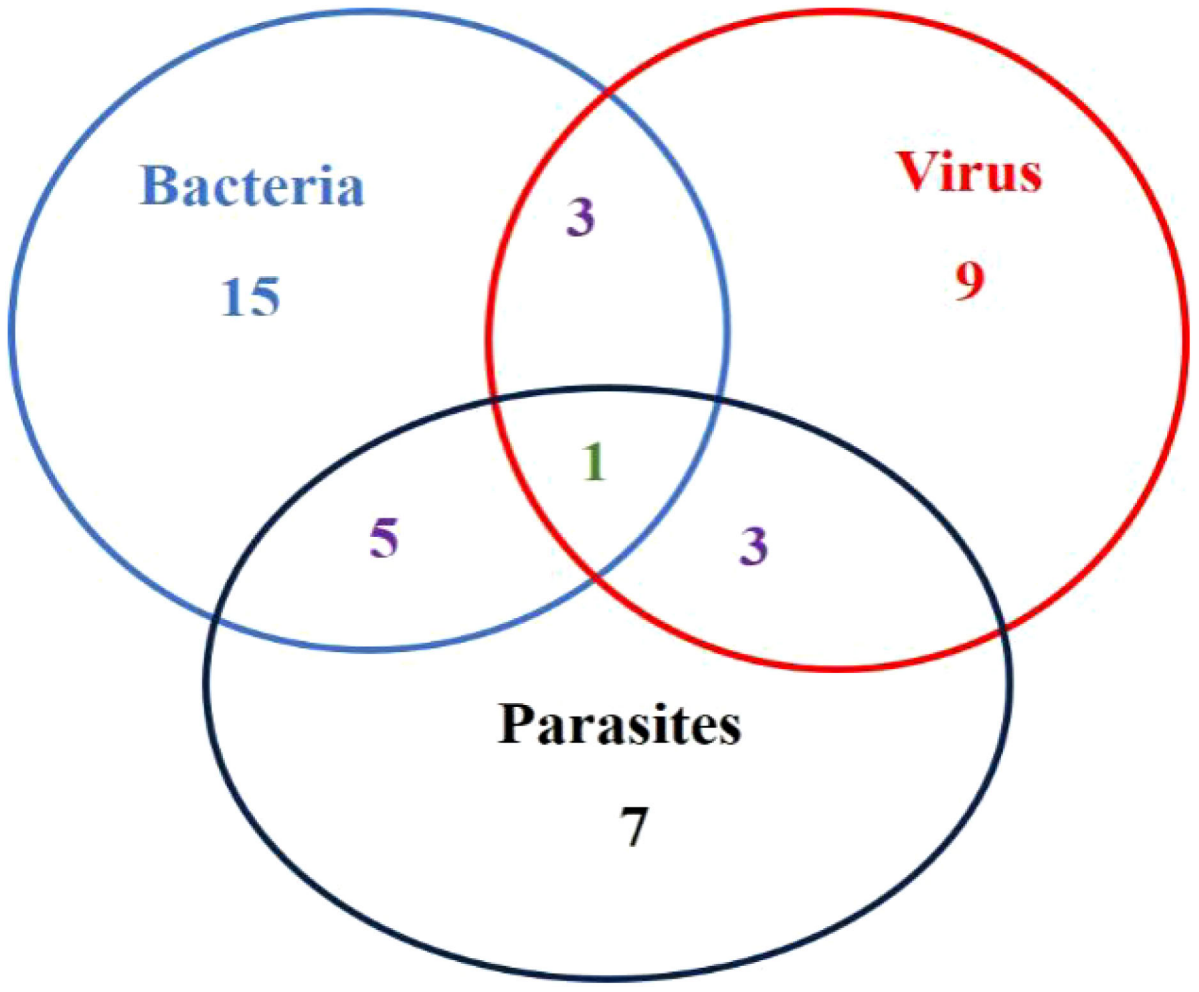
Venn diagram of various pathogens in *L. crocea*.

In this study, a total of fifty-five *L. crocea* were collected, of which 78.18% (43/55) *L. crocea* were found to have disease-causing pathogens. The other *L. crocea* (21.82%) did not identify any pathogens, which possibly due to injuries in farming or nutritional metabolic disorders leading to death. Among *L. crocea* infected by the pathogens, twenty-four diseased fish had bacterial infections, sixteen fish was parasitic infection, and sixteen fish was virus infection. The overall proportions of various pathogens are shown in **Figure 1**. Those includes five diseased fish co-infected with bacteria and parasites, three fish co-infected with bacteria and viruses, and three fish co-infected with parasites and viruses. In addition, one *L. crocea* was infected by mixture of viruses, bacteria, and parasites (**Figure 2**).

### 3.2 Bacterial isolation

**Figure 3:**
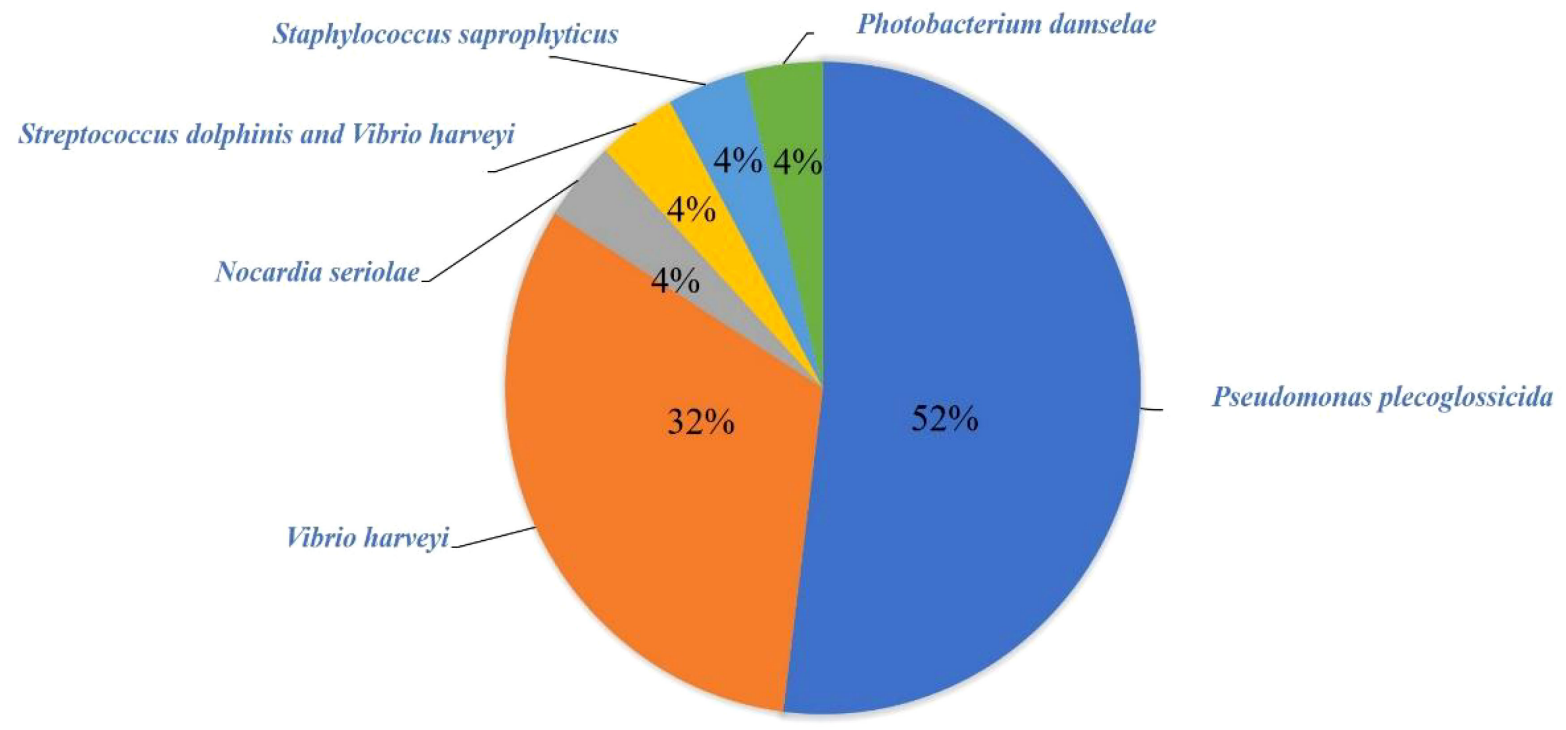
Species and abundance of bacteria.

A total of 25 pathogenic bacteria were isolated from those above 24 diseased *L. crocea*. These pathogenic bacteria are mainly *Pseudomonas plecoglossicida* and *Vibrio harveyi*, accounting for 52% (13/25) and 32% (8/25) of the total bacterial strains, respectively (**Figure 3**). Beyond that, there is one *L. crocea* infected both by *Streptococcus iniae* and *V. harveyi*. Moreover, three other bacteria were also detected, they were *Nocardia seriolae*, *Staphylococcus saprophyticus*, and *Photobacterium damselae*, respectively (**Table 2**).

**Table 2:** Statistics of bacterial infection of *L. crocea*.

No.	Bacterial species	The number of *L. crocea* (tails)
1	*P. plecoglossicida*	12
2	*V. harveyi*	8
3	*N. seriolae*	1
4	*Co-infection of S. iniae and V. harveyi*	1
5	*S. saprophyticus*	1
6	*P. damselae*	1

### 3.3 Parasite detection statistics

**Figure 4:**
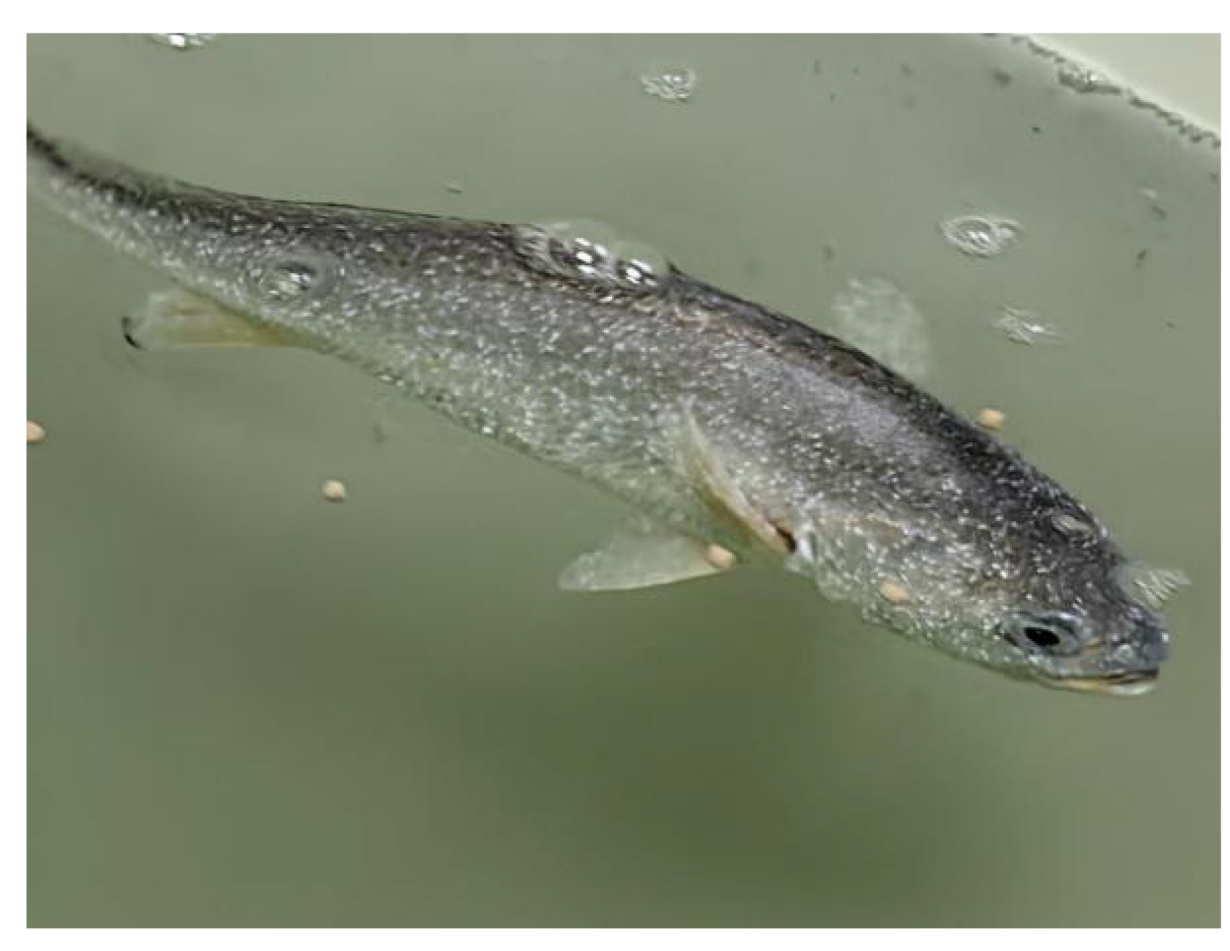
*C. irritans* infected *L. crocea*.

**Figure 5:**
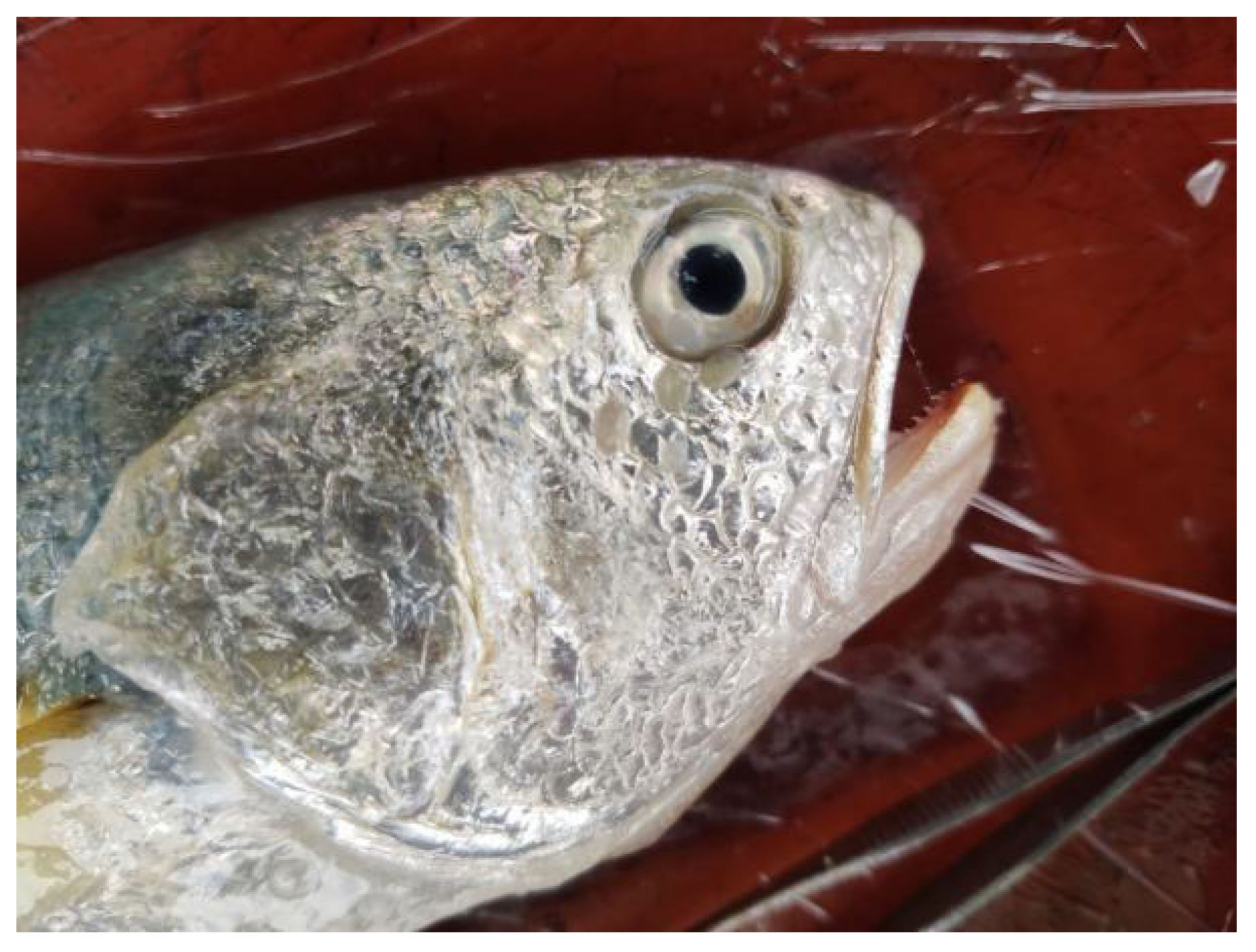
*N. girellae* infected *L. crocea*.

**Figure 6:**
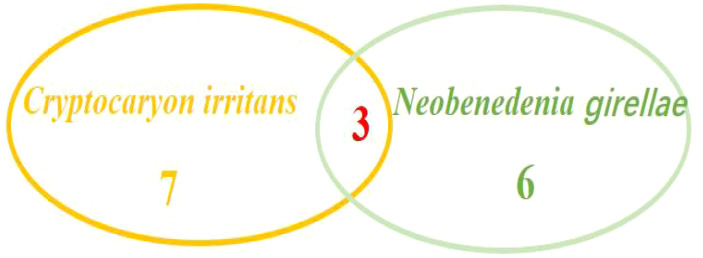
Venn diagram of parasites in *L. crocea*.

Among the above 55 *L. crocea*, a total of 16 were infected with parasites. Among them, 7 fish were infected with *Cryptocaryon irritans*(**Figure 4**), 6 fish were infected with *Neobenedenia girellae* (**Figure 5**), and 3 fish had mixed infections of *C. irritans* and *N. girellae* (**Figure 6**).

### 3.4 Mixed infections of parasites, bacteria, and virus

**Figure 7:**
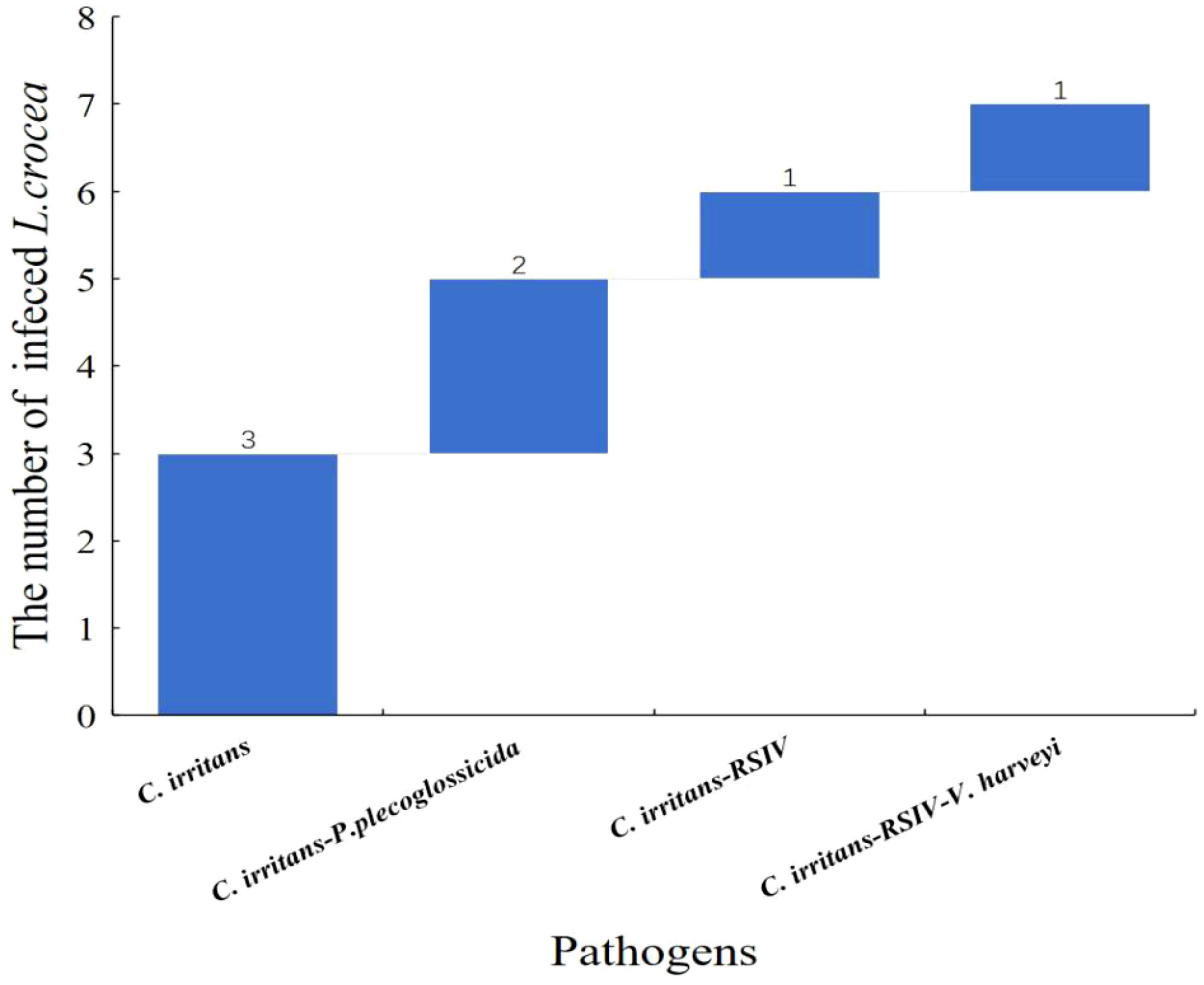
Waterfall diagram of *C. irritans* and its co-infection with the other pathogens in *L. crocea. C. irritans* means only the *C. irritans* infection in *L. crocea*; *C. irritans* - P*. plecoglossicida* means the co-infection of *C. irritans* and *P. plecoglossicida*; *C. irritans* -RSIV means the co-infection of *C. irritans* and RSIV; *C. irritans*- RSIV - V*. harveyi* means the co-infection of *C. irritans*, RSIV and *V. harveyi*.

Among the seven *C. irritans* infected *L. crocea* mentioned above, three *L. crocea* were only infected by *C. irritans*, two *L. crocea* had mixed infections of *C. irritans* and *P. plecoglossicida*, one *L. crocea* had mixed infections of *C. irritans* and RSIV, and one *L. crocea* had mixed infections of three pathogens including *C. irritans*, RSIV, and *V. harveyi* (**Figure 7**).

**Figure 8:**
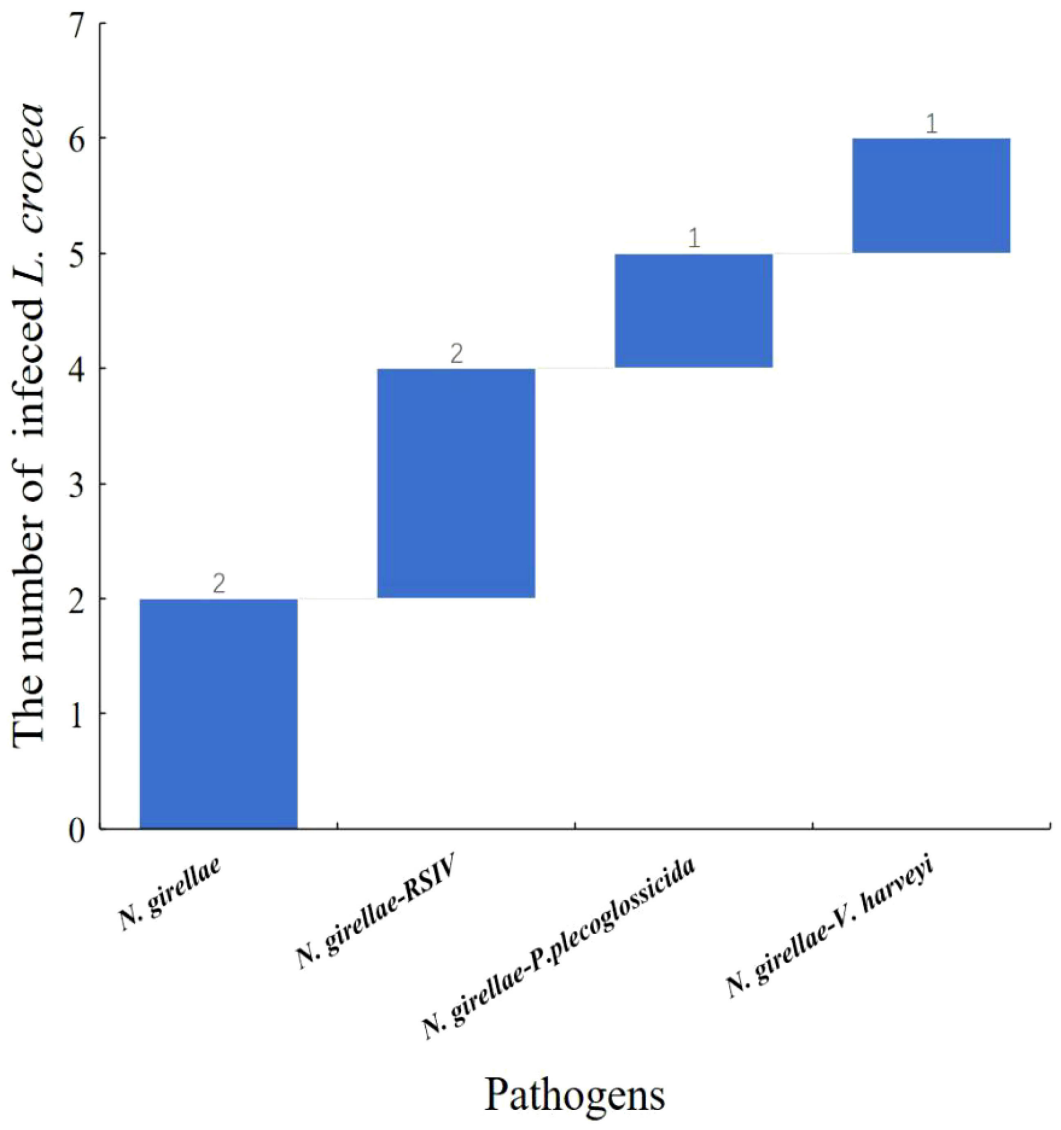
Waterfall diagram of *N. girellae* and its co-infection with the other pathogens in *L. crocea. N. girellae* means only the *N. girellae* infection in *L. crocea*; *N. girellae-*RSIV means the co-infection of *N. girellae* and RSIV; *N. girellae - P. plecoglossicida* means the co-infection of *N. girellae* and *P. plecoglossicida*; *N. girellae* - V*. harveyi* means the co-infection of *N. girellae* and *V. harveyi.*

Among the six *N. girellae* infected *L. crocea* mentioned above, two *L. crocea* were only infected with *N. girellae*, two *L. crocea* had mixed infections with *N. girellae* and RSIV. In addition, one *L. crocea* was mixed infected by *N. girellae* and *P. plecoglossicida*, and one *L. crocea* was mixed infected by *N. girellae* and *V. harveyi* (**Figure 8**).

**Figure 9:**
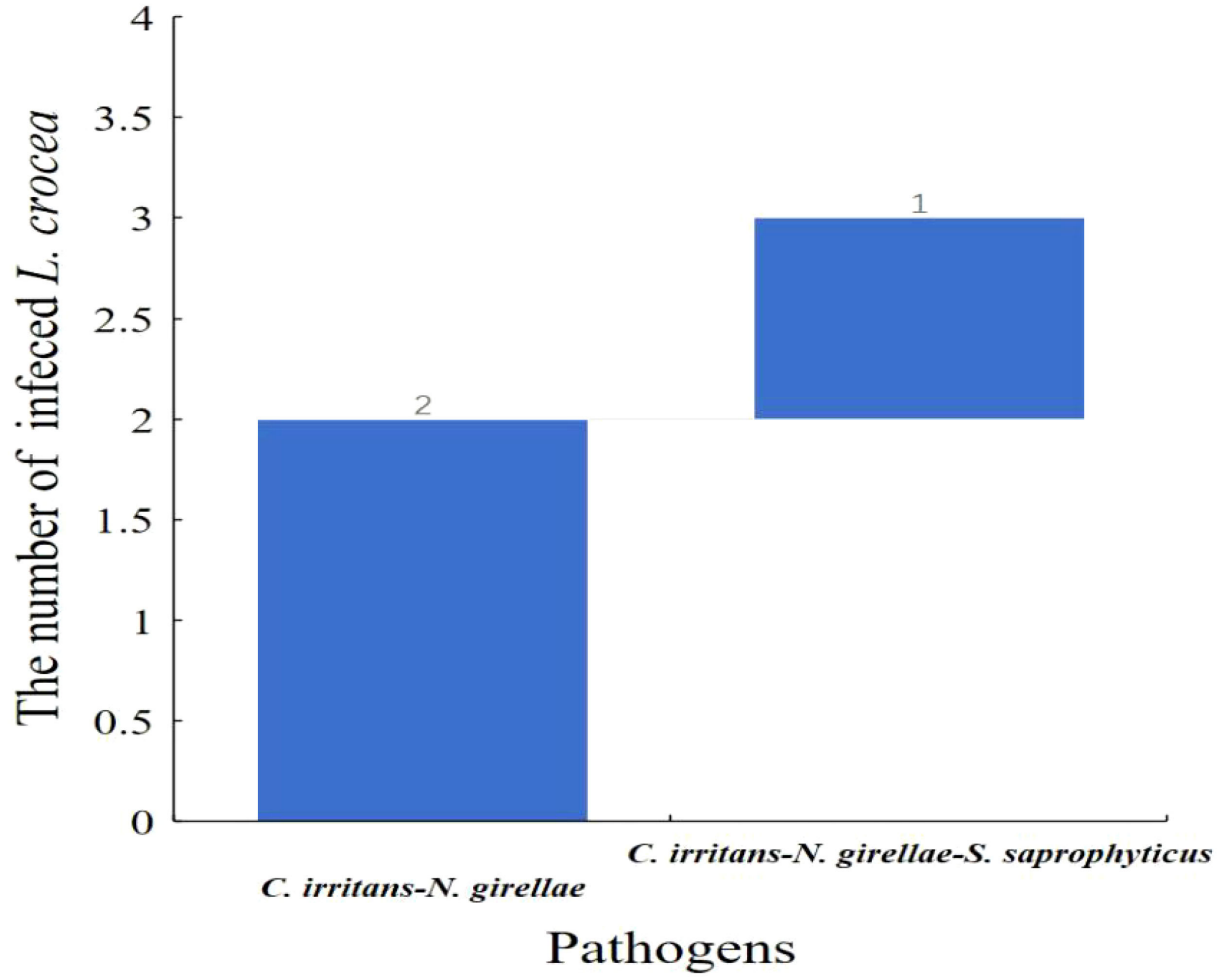
Waterfall diagram of two parasites (*C. irritans* and *N. girellae*) and co-infection with the other pathogens in *L. crocea. C. irritans* -*N. girellae* means the co-infection of *C. irritans* and *N. girellae*; *C. irritans* -*N. girellae- S. saprophyticus* means the co-infection of *C. irritans*, *N. girellae* and *S. saprophyticus*.

Moreover, among the three *L. crocea* with mixed infections of *C. irritans* and *N. girellae*, two *L. crocea* were parasite infections, and one had mixed infections of the two parasites (*C. irritans* and *N. girellae*) with *S. saprophyticus* (**Figure 9**).

### 3.5 Virus detection statistics

**Figure 10:**
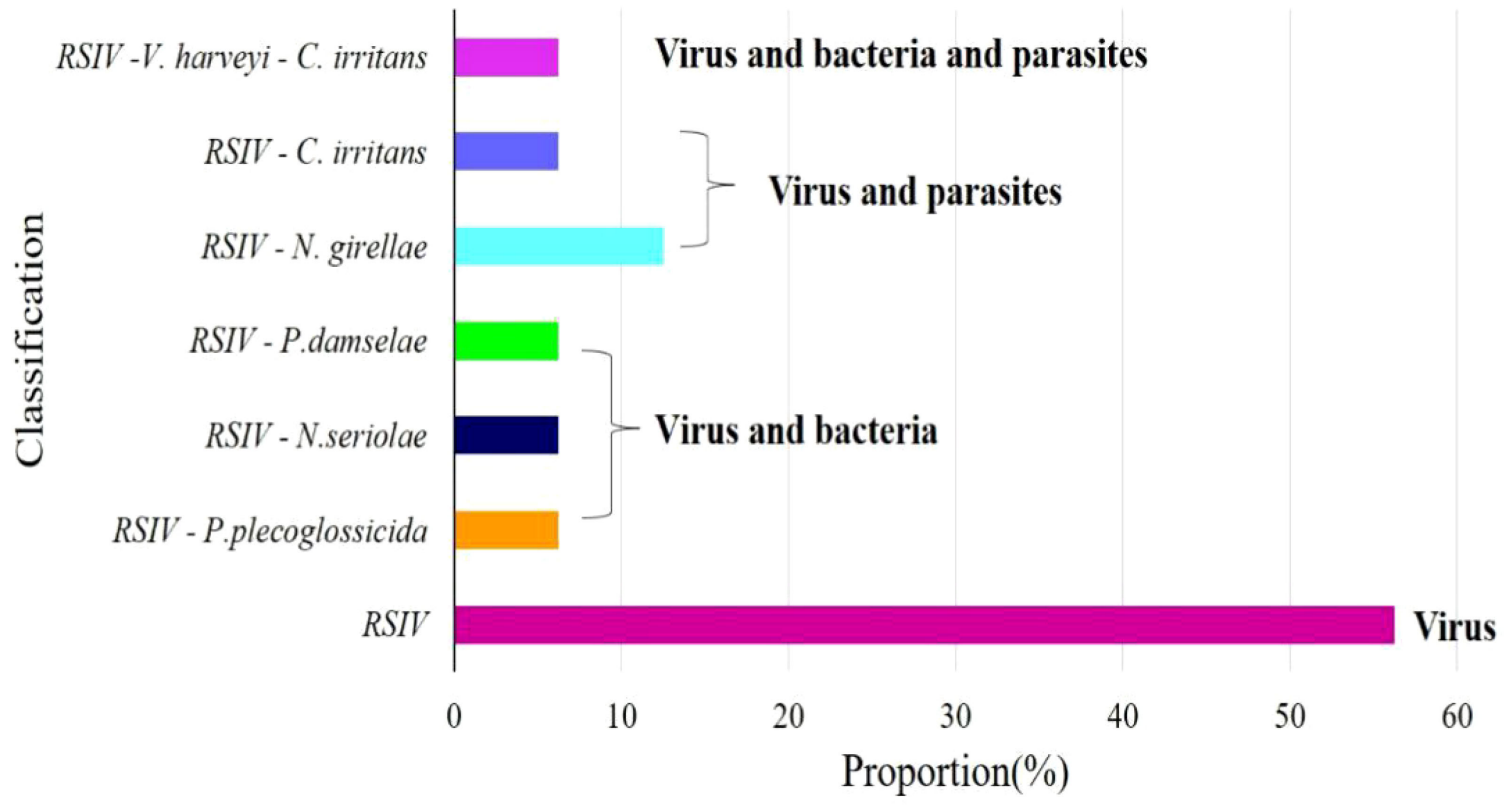
Virus detection ratio bar chart. RSIV means only the virus infection in *L. crocea*; RSIV-*P. plecoglossicida* means the co-infection of RSIV and *P. plecoglossicida*; RSIV-*N. seriolae* means the co-infection of RSIV and *N. seriolae*; RSIV- *P. damselae* means the co-infection of RSIV and *P. damselae*; RSIV-*N. girellae* means the co-infection of RSIV and *N. girellae*; RSIV-*C. irritans* means the co-infection of RSIV and *C. irritans*; RSIV-*V. harveyi* -*C. irritans* means the co-infection of RSIV and *V. harveyi* and *C. irritans*.

The virus ISKNV, RSIV, and NNV can infect *L. crocea*. In previous epidemiological surveys of aquaculture diseases, these three viruses were detected in *L. crocea*. However, only RSIV was detected in this investigation, indicating a higher prevalence of RSIV in Ningbo *L. crocea* farming area. Among the samples, sixteen fish were carriers of this pathogen, of which 56.25% (9/16) were infected with the virus alone, and 43.75% (7/16) were co-infected with other pathogens. Among them, one fish was infected with *P. plecoglossicida*, *Nocardia seriolae*, and *Photobacterium damselae*, respectively. And one fish was infected with *N. girellae* and *C. irritans* parasites, respectively. In addition, there was one *L. crocea* co-infected with RSIV, *V. harveyi* and *C. irritans* (**Figure 10**, **Table 3**).

**Table 3:** Virus and co-infection with the other pathogens in *L. crocea*.

No.	Virus species	The number of *L. crocea* (tails)
1	RSIV	9
2	RSIV and *P. plecoglossicida*	1
3	RSIV and *N. seriolae*	1
4	RSIV and *P. damselae*	1
5	RSIV and *N. girellae*	2
6	RSIV and *C. irritans*	1
7	RSIV, *C. irritans* and *V. harveyi*	1

### 3.6 Pathogen detection status in different month

As shown in **Table 4**, nine types pathogens were isolated. *P. plecoglossicida* infection was mainly distributed from April to June and October. *V. harveyi* infections in *L. crocea* usually occur from June to August and October. Bacteria such as *N*. *seriolae*, *S. saprophyticus*, *S. iniae*, and *P. damselae* mainly happen in October. In addition, parasites such as *C. irritans* and *N. girellae* are prevalent during May to October and August to October, respectively. In terms of viral pathogens, RSIV are spread in May, July, August, September, and October. Knowing the seasonal time of common pathogen outbreaks is conductive and meaningful in disease prevention and treatment, improving the production of *L. crocea*.

**Table 4:** Pathogen species isolated in *L. crocea* in different month.

Pathogens	April	May	June	July	August	September	October
	Water temperature(°C)						
	16.3	20.5	26.5	29.0	30.2	29.2	24.0
*P. plecoglossicida*	+	+	+	0	0	0	+
*V. harveyi*	0	0	+	+	+	0	+
*N.seriolae*	0	0	0	0	0	+	0
*S. saprophyticus*	0	0	0	0	0	0	+
*S. iniae*	0	0	0	0	0	0	+
*P. damselae*	0	0	0	0	0	0	+
*C. irritans*	0	+	+	+	+	+	+
*N. girellae*	0	0	0	0	+	+	+
RSIV	0	+	0	+	+	+	+

## 4 Discussion

Marine aquaculture is an indispensable part of China’s fisheries and an important part of the national economy. *L. crocea* is a major aquaculture fish in Fujian, Zhejiang, and Guangdong provinces in China. The booming development of the aquaculture industry of *L. crocea* has directly or indirectly driven the coastal rural economy and promoted the employment of coastal labor. Nevertheless, there are a series of problems, such as the quality decline and serious aquaculture sea pollution, that lead to low fish farming production and high farming risks. Improving the quality of *L. crocea* and reducing the risk of breeding, needs to be addressed urgently. Fish disease has become one of the main obstacles to the development of the *L. crocea* industry.

At present, the diseases in *L. crocea* aquaculture industry are mainly caused by bacterial, viral, and parasitic pathogens. The bacteria such as *N. seriolae* (Wang et al., 2008),*V. harveyi* (Lin et al., 1999; Ji et al., 2004), *V. parahaemolyticus, P. plecoglossicida* (Yan et al., 2022)*, S. iniae and P. damselae* (Zhang et al., 2012; Ge et al., 2022), all can infect *L. crocea*. In this study, bacterial pathogens *P. plecoglossicida, V. harveyi, N. seriolae, S. iniae and P. damselae all were* identified in *L. crocea*. In addition, *S. saprophyticus* was detected in diseased *L. crocea*, which is also the first report of isolation in diseased *L. crocea*.

The statistical analysis of bacterial infection demonstrated that *P. plecoglossicida* and *V. harveyi* accounted for 52% and 32% of the total strains, respectively, indicating that these two bacteria were the main bacterial species that harmed the culture of *L. crocea* in Ningbo. In addition, a mixed infection that *S. iniae and V. harveyi* co-infected *L. crocea* was verified, indicating the complexity of pathogenic infection. By investigating infection seasons of bacteria, we find *P. plecoglossicida* mainly happened from April to June and October. Previous research showed the infection occurs during winter and into the next spring, when the water temperature is lower than 20° (Qiu et al., 2012). The infection developed clinical signs of *L. crocea* at 16–19°C but not at 24–27°C (Mao et al., 2024), which are consistent with this epidemiological investigation. In addition, a variety of bacteria such as *S. saprophyticus*, *S. iniae*, and *P. damselae* were isolated from samples taken in October, which may be related to the sudden change in water temperature and the decline in fish resistance to disease.

Compared with bacterial diseases, some parasitic diseases are more prevalent and more devastating. In marine fish farming, *N. girellae* (Umeda and Hirazawa, 2004), *Amyloosinium ocellatum* (He, 2013), *C*. *irritans* (Liu et al., 2022; Zhan et al., 2023) were common parasites. The wounds on the body’s surface caused by parasites are easily co-infected by bacteria, leading to severe inflammation, scale shedding, muscle ulcer perforation, excessive mucus secretion, inhibition of breathing, and finally the death of the fish. In this study, *C. irritans* and *N. girellae* are two parasites identified and accounted for nearly 30% samples. Investigation showed that *C. irritans* occurred from May until October, indicating huge harm on *L. crocea* farming. *C. irritans* mainly parasitize the gills and body surface of fish, forming a small “white spots”. Due to strong environmental adaptability and high fatality rate, the infection caused by *C. irritans* has become the most destructive parasitic disease in seedlings and adult fish (Bai et al., 2023).

In the early stage of infection, symptoms such as increased mucus on the body surface and gills, and decreased appetite appear. After that, excessive mucus secretion will wrap the fish body, and fish will swim abnormally and rubbing the pool wall or cage. In the later stage of infection, fish shows difficulty breathing with sluggish behavior, and presents more obvious and thick white spots on the body’s surface, and finally lead to the death. When infected by *C. irritans*, the wounds caused by rubbing the wall of the pond can be easily secondarily infected by bacteria, which was confirmed by some scholars by isolating the bacteria from the fish body in the fish infected by *C. irritans* (Liu et al., 2012). In this study, 43% of *L. crocea* infected with *C. irritans* were co-infected with *P. plecoglossicida* or *V. harveyi*, which also confirmed the susceptibility of parasitic infection to bacterial secondary infection. *N.girellae* was one important pathogen of *L. crocea*, which was parasitic on the body surface, fins, and gills, with high mortality near 80% during the epidemic season in July to October (Yang et al., 2001).In this study, *N. girellae* infection mainly occurs from August to October, which is basically similar to the previous reports. However, pathogenic species analysis found *N. girellae* and *C. irritans* co-infected *L. crocea*. Even, a certain proportion of parasites had mixed infections with *Staphylococcus saprophyticus*, namely three pathogens co-infection, indicating that some breeding environments were harsh and disease outbreaks were extremely serious.

Iridoviruses (RSIV), the causative agents of serious systemic diseases among cultured, ornamental, and wild fish, have been identified from more than 20 fish species in recent years (Qin et al., 2002). Mortalities of fish caused by systemic RSIV infection reaching 30–100% were observed (Chen et al., 2023a). RSIV had caused high mortality in *L. crocea* during last few years, and no effective prevention measure was used to control this RSIV disease until now. Development of a rapid and valid method was of great importance for the early diagnosis of RSIV infection and controlment of RSIV disease (Chen et al., 2023b). In this study, the proportion of *L. crocea* carried RSIV has reached 29%, while infectious ISKNV and NNV were not detected, indicating that the RSIV is more prevalent and harmful to the survival of *L. crocea*.

At present, there is no effective prevention and control method for viral diseases in *L. crocea*. Chemical drugs still dominate in bacterial and parasitic diseases. However chemical drugs have limitations that will cause serious drug resistance in pathogenic bacteria. For example, *N*. *seriolae* and *P. plecoglossicida* in the early infection phase hard to detect. Farmers use more antibiotics for preventing these pathogens in advance, which causes drug residue issues, posing serious threats to the safety of aquatic products. In the aspect of parasitic prevention and control, chemicals such as copper sulfate and formaldehyde are currently used. However, the *L. crocea* breeding environment is mostly an open water system, the entire aquatic ecosystem is seriously polluted and the effect of drug application is very poor. Given the potential problems of chemical drugs in the prevention and treatment of diseases in *L. crocea*, it is imperative to develop safe and effective prevention and treatment techniques.

## Data availability statement

The raw data supporting the conclusions of this article will be made available by the authors, without undue reservation.

## Ethics statement

The use of experimental animals and the study protocol were reviewed and approved by the Ningbo Ocean and Fisheries Research Institute. The studies were conducted in accordance with the local legislation and institutional requirements. Written informed consent was obtained from the owners for the participation of their animals in this study.

## Author contributions

SX: Writing – review & editing, Writing – original draft. MG: Writing – review & editing, Writing – original draft. JF: Writing – original draft. XW: Writing – review & editing. HT: Writing – review & editing. ZL: Writing – review & editing. GT: Writing – review & editing.
